# Comparison of socioeconomic and psychosocial profiles between Brazilian and Swedish women with temporomandibular disorders

**DOI:** 10.1038/s41598-026-47939-z

**Published:** 2026-04-08

**Authors:** Marlon Ferreira Dias, Hajer Jasim, Daniela Aparecida de Godoi Gonçalves, Malin Ernberg

**Affiliations:** 1 Department of Dental Materials and Prosthodontics, School of Dentistry, São Paulo State University (UNESP), Araraquara, Brazil; 2 Division of Oral Rehabilitation, Department of Dental Medicine, Karolinska Institutet, SE 14104 Huddinge, Sweden; 3 Department of Orofacial Pain and Jaw Function, Public Dental Services, Folktandvården Stockholm, Eastmaninstitutet, SE-102 31 Stockholm, Sweden

**Keywords:** Demography, Pain, Psychosocial functioning, Temporomandibular joint disorders, Diseases, Health care, Medical research, Psychology, Psychology, Risk factors, Signs and symptoms

## Abstract

Temporomandibular disorders (TMD) are highly affected by psychological factors and may also be associated with socioeconomic disadvantages. This study aimed to investigate whether psychosocial and demographic factors of women with TMD differ between two countries with different socioeconomic development levels. 300 women with myogenous TMD in Brazil (*n* = 141) and Sweden (*n* = 159) were characterized regarding sociodemographic and psychological data and were clinically examined according to Axis I and II of the diagnostic criteria for temporomandibular disorders. The cohorts were compared regarding demographics, characteristic pain intensity, pain interference, the presence of widespread pain, oral parafunctions, limitations in jaw function, and symptoms of depression, anxiety, and non-specific physical symtoms. Data analysis included Mann-Whitney U-Test, Chi-square test, and Fisher’s exact test (*p* < 0.05). Bonferroni test for multiple comparisons was also applied (*p* < 0.01). Sociodemographic data revealed differences in body mass index (BMI), marital status, education level, and employment status (*p* < 0.05), with higher BMI, greater frequency of single marital status, and higher education level in the Brazilian cohort. The Swedish cohort showed higher levels of pain interference, greater limitations in jaw function, and more frequent widespread pain (*p* < 0.05), while anxiety levels were higher in the Brazilian cohort (*p* < 0.05). Characteristic pain intensity, oral parafunctions, depression, and non-specific symptoms did not differ between cohorts (*p* > 0.05). There were both differences and similarities in psychological and sociodemographic factors between the two TMD cohorts in Brazil and Sweden. The comprehensive assessment of TMD diagnoses, psychosocial and demographic characteristics may help to guide TMD care and future research in countries with different socioeconomic contexts.

## Introduction

Temporomandibular disorders (TMD) are highly prevalent public health problems that involve painful alterations in the masticatory muscles, temporomandibular joints, and surrounding structures^2^. Among all chronic orofacial painful conditions, TMD pain is the most common non-odontogenic type of pain^1^, affecting approximately 5–12% of the general population, with a higher prevalence in women than in men^3,4^.

The clinical manifestation of TMD can negatively impact the quality of life, and the symptoms may include joint sounds, muscle and/or joint pain, mouth opening limitation and deviation/deflection in the path of opening^5,6^. Its etiology is considered multifactorial, including the presence and frequency of parafunctional habits, genetic, hormonal, stress, and sex related factors. Additionally, various physiological and psychosocial aspects may contribute to its onset^7–9^.

Moreover, symptoms of anxiety and depression have been associated with increased TMD pain intensity and may play an important role in its maintenance and prognosis^10–13^. Also, individuals with chronic TMD are more likely to report higher levels of somatic awareness and pain catastrophizing than those free of TMD^14^. Although socioeconomic factors, which differ among various populations, can influence psychological factors^15^, there is a lack of research examining these aspects in countries with different cultural background and socioeconomic development.

Cross-cultural differences play an important role in pain perception and expression of chronic pain conditions in general, including TMD^16,17^. In this context, comparisons across different TMD patient populations may provide an important insight into the influence of culture and ethnicity on this condition^18^. Previous studies have demonstrated variations in TMD characteristics among distinct populations^14,19^. Furthermore, ethnicity has been associated with these differences. For example, variations in TMD symptoms, personality, and psychosocial profile have been reported among Malay, Chinese, and Indian patients in a recent study^20^. Another risk factor for TMD is low income, and a systematic review with meta-analysis reported a significant association of these aspects with TMD prevalence^21^. However, few studies have investigated whether a country’s socioeconomic development, i.e. the economy, technological development, and standards of living, influences sociodemographic factors and psychological distress associated with TMD.

To clarify this association, we conducted a retrospective cross-sectional study to investigate whether psychosocial and demographic factors differ among women with TMD from two countries with different socioeconomic development levels. The countries included were Sweden, a high-income welfare country, and Brazil an upper-middle income country with greater socioeconomic disparities. The null hypothesis was that there is no difference between Brazilian and Swedish TMD patients regarding psychological distress and sociodemographic data.

## Methods

### Study design and participants

This retrospective study was conducted with a sample of women from Brazil and Sweden. All methods were performed in accordance with the Declaration of Helsinki. The inclusion criteria were women aged between 18 and 50 years with a TMD myalgia diagnosis or myofascial pain with referral according to the Diagnostic Criteria for TMD (DC/TMD)^4^. Exclusion criteria for both samples included male patients, individuals with an isolated diagnosis of arthralgia, and individuals younger than 18 or older than 50 years of age.

In Brazil, participants were recruited from individuals with TMD who sought treatment at the Araraquara School of Dentistry, São Paulo State University (Unesp), their companions, and through social media. The data were collected between March 1, 2024 to June 1, 2025. Individuals who agreed to participate were instructed to sign the informed consent form, approved by the Human Research Ethics Committee of Araraquara School of Dentistry, UNESP (CAAE: 68830323.8.0000.5416). The patient data was transferred to an Excel database that was pseudonymized.

The Swedish cohort included individuals who had been referred to the Specialist clinic for orofacial pain and jaw function, Karolinska Institutet, Huddinge, Sweden because of orofacial pain. Self-reported data were collected electronically between March 1, 2019 to December 31, 2023 and downloaded to an Excel database. The patients’ DC/TMD diagnoses were then retrieved from the patient’s medical records and added to the database whereafter it was pseudonymized. In the electronic questionnaire patients were asked to provide consent that their data may be used in research studies. Only data from patients who have provided consent were included in the database. Incomplete or duplicate questionnaires were excluded from the database. The use of data for this study was approved by the Swedish Ethical Review Authority (reg nr 2025-02802-01; 250514).

### General methodology

All participants had completed the DC/TMD Axis II questionnaire and were clinically examined according to the Axis I^4,22^.

#### Sociodemographic data

Data regarding age, marital status, employment, and education level were collected from all participants to characterize the study sample and to enable comparisons between countries.

#### Body mass index

The Body Mass Index (BMI) of each participant was calculated from height and weight. In Brazil, data were obtained through direct measurements taken by a researcher, whereas in Sweden, participants self-reported this information. The results were dichotomized as BMI < 25 and BMI ≥ 25, based on the limits set by the World Health Organization^23^.

### Pain features and interference

#### Graded chronic pain scale

To evaluate the severity of pain and disability related to TMD, we used the Graded Chronic Pain Scale – version 2 (GCPS)^24^. The GCPS includes the measurement of the Characteristic Pain Intensity (CPI) and the Disability Points (DP) for the number of days with disability. Herein, we compared the CPI, the DP scores, and the total GCPS score between the groups. For the analysis, the classification of chronic pain interference was made according to the following grades: Grade 0: No pain; Grade I: Low intensity pain, without disability; Grade II: High intensity pain, without disability; Grade III: Moderately limiting; Grade IV: Severely limiting^24^.

#### Widespread pain

The widespread pain index (WPI) was used for the assessment of widespread pain in patients from Sweden^25^. The WPI assesses bodily pain in 19 specified locations, and widespread pain is defined as the presence of pain in at least 4 out of 5 bodily regions (right upper, left upper, right lower, left lower, and axial)^25^. In Brazil, pain drawings were used to assess widespread pain in a similar manner, i.e. pain in 4 out of the 5 regions on the drawings were defined as presence of widespread pain.

### Psychological distress

Psychosocial distress was evaluated using the instruments that compose the DC/TMD Axis II. The results were interpreted following the Scoring Manual for Self-Report Instruments of the DC/TMD^22^.

#### Anxiety and depression

The anxiety symptoms experienced in the past two weeks were assessed using the Generalized Anxiety Disorder-7 (GAD-7) questionnaire^26^. This instrument consists of 7 questions, which are scored from 0 to 3, with a total score ranging from 0 to 21. Scores greater than or equal to 10 indicate the presence of anxiety symptoms^26^. The symptoms of depression were assessed using the Patient Health Questionnaire-9 (PHQ-9), which consists of 9 questions with four possible responses (0–3), and the total score ranging from 0 to 27^27^. Scores greater than 10 indicate the presence of symptoms of depression^27^.

#### Non-specific physical symptoms

These were assessed with the Patient Health Questionnaire-15 (PHQ-15), which comprises a list of 15 somatic symptoms^28^. Each symptom is scored from 0 (“not bothered”) to 2 (“very bothered”). Items are scored by adding the individual responses and the total sum score is computed. Scores of 5, 10, and 15 represent cut-points for low, medium, and high physical symptoms, respectively^28^.

### Jaw functional limitation

Jaw Functional Limitation Scale (JFLS-20) was applied to assess the functional status of the masticatory system^29^. This instrument comprises 20 activities covering three areas: chewing, mobility and communication, graded from 0 “no limitation” to 10 “severe limitation”^29^. At present, no established cut-off point has been provided, but the higher the JFLS-20 score, the more severe the jaw functional limitation.

### Oral behaviors

To identify the frequency of oral behaviors the Oral Behaviors Checklist (OBC) was applied^30^. In this 21-item self-report questionnaire, the individuals were asked to report frequency of oral behaviors during day and night over the past month, using responses of none of the time, < 1 night/month, 1–3 nights/month, 1–3 nights/week, and 4–7 nights/week (score range 0–4). This yields a total score ranging from 0 to 84^30^. For analysis, total scores of (0–16) represent normal range, (17–24) moderate risk, and (25–62) high risk of TMD.

### Statistical analysis

All analyses were performed using IBM SPSS Statistics software version 29.0.0.0 (SPSS Inc., Chicago, IL, USA). The Kolmogorov-Smirnov test was applied to assess the normality of the continuous variables. Data are presented as mean (SD) or median and interquartile range (IQR) for continuous variables and percentage (n, %) for categorical variables.

Continuous and normally distributed data was analyzed with t-test. The total scores from the instruments in the Axis II questionnaire may seem like continuous data. However, they are composed of a sum score based on individual scores for the various questions assessed with a verbal scale that are given an ordinal number. We therefore consider parametric statistics inappropriate for analysis of these data. Thus, the non-parametric Mann-Whitney U-test was used to analyze these data.

Categorical variables such as marital status, education level, employment status, WPI, TMD diagnoses, and GCPS were analyzed using the Chi-square test or Fisher’s exact test, as appropriate. The chi-squared test was applied assuming the sample is large, while the Fisher’s exact test was used for smaller samples, particularly when more than 20% of cells had expected frequencies < 5. To account for multiple comparison when analyzing subcategories of demographic variables and GCPS Bonferroni correction was applied, with significant threshold of *p* < 0.01. For all other analysis a significance level of *p* < 0.05 was considered statistically significant.

A sensitivity analysis was performed in G*Power for the Wilcoxon-Mann-Whitney test for two independent groups. Considering Brazil (*n* = 149) and Sweden (*n* = 151), two-tailed α = 0.05, and 95% power, the study was able to detect effect sizes of d ≥ 0.42, indicating sensitivity to detect effects of approximately small-to-moderate magnitude or larger.

## Results

**Fig. 1:**
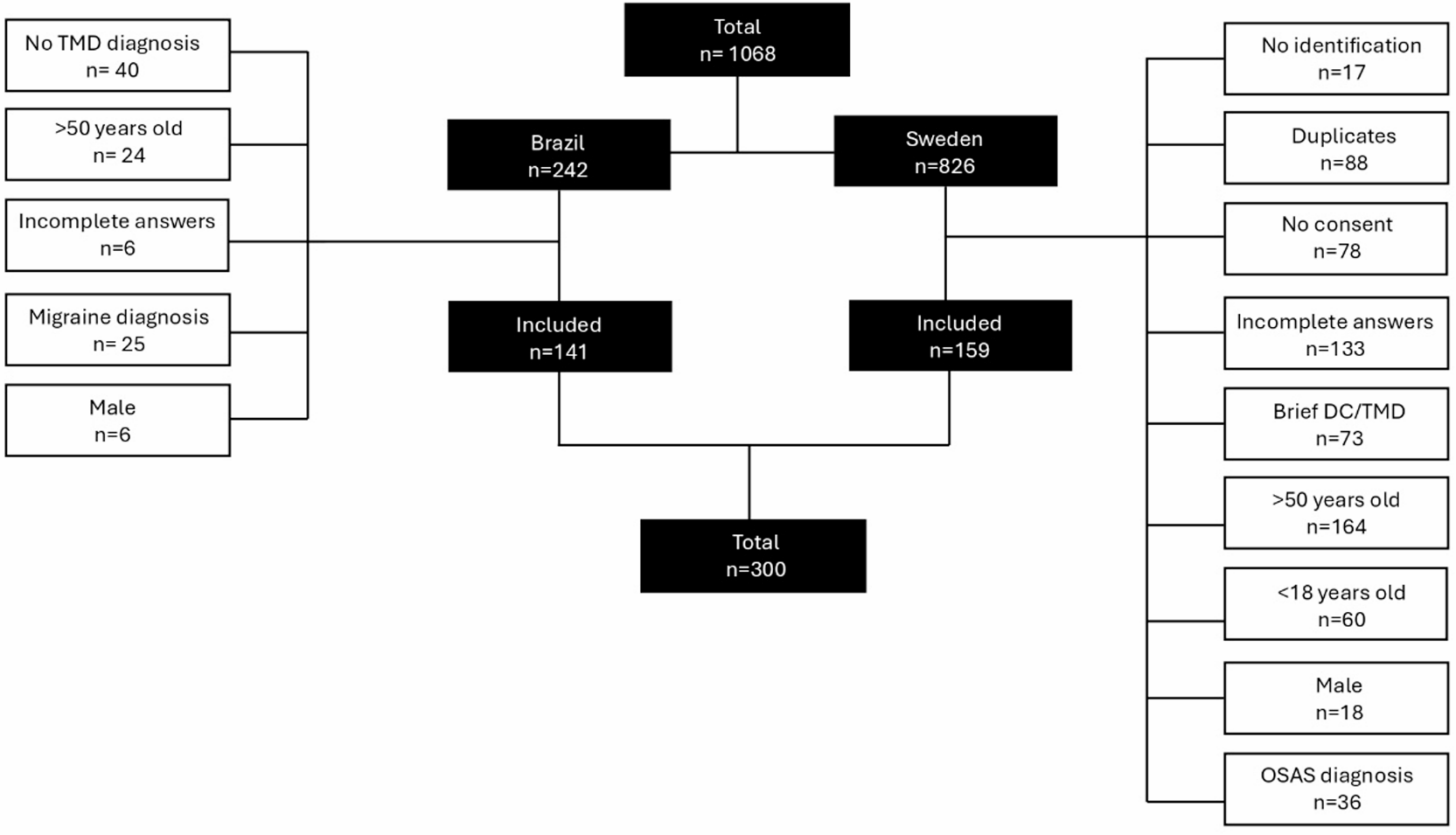
Flowchart of participant selection according to the inclusion and exclusion criteria. TMD: Temporomandibular Disorders; DC/TMD: Diagnostic Criteria For Temporomandibular Disorders.

A total of 1,068 clinical records were assessed in Brazil and Sweden. Of 242 participants from Brazil, 141 were included according to the inclusion criteria. In Sweden, out of 826 participants, 159 were included following the same criteria. The final sample included 300 women aged 18 to 50 years (Fig. 1).

When analyzing data, we found that age and BMI were not normally distributed, hence, non-parametric tests were used for all data.

Demographic data showed a higher median BMI in the Brazilian cohort compared to the Swedish cohort, as well as significant differences in marital status and education level. Regarding employment status, only the retired and sick leave categories demonstrated significant differences, with a higher number of retired in the Brazilian cohort and a higher number of patients on sick leave in the Swedish cohort (Table 1).

**Table 1:** Demographic description of the overall sample.

		Brazil	Sweden	*p*-value	Effect size
Sample size		141	159		
Age		30 (13.0)	27 (20.0)	0.417	-0.054
BMI		25 (6.4)	23 (5.5)	**0.007**	**-0.182**
Marital status				**< 0.001**	**0.538**
Single		92 (65.2)	34 (21.4)	**< 0.001**	**0.444**
Married		43 (30.5)	69 (43.4)	0.116	0.133
Divorced		4 (2.9)	1 (0.6)	0.953	0.086
Other		0 (0)	53 (33.3)	**< 0.001**	**0.436**
Missing data		2 (1.4)	2 (1.3)	1.000	0.006
Education level				**< 0.001**	**0.417**
Primary school		0 (0)	18 (11.3)	**< 0.001**	**0.238**
High school		64 (45.4)	53 (33.3)	0.168	0.123
University		77 (54.6)	57 (35.9)	**0.005**	**0.188**
Other		0 (0)	9 (5.7)	0.019	0.166
Missing data		0 (0)	22 (13.8)	**< 0.001**	**0.265**
Employment*					
Employed		88 (59.1)	90 (53.2)	0.371	0.051
Unemployed		6 (4.1)	16 (9.5)	0.051	0.113
Student		48 (32.2)	54 (31.2)	0.949	0.003
Retired		7 (4.6)	0 (0)	**0.005**	**0.163**
Sick leave		0 (0)	9 (5.3)	**0.003**	**0.167**

BMI: Body Mass Index; IQR: interquartile range; Other: for marital status, relationship/living arrangements not captured by the predefined categories (e.g., non-cohabiting relationships or living with parents); for education level, educational pathways not classified within the predefined categories (e.g., folk high school education or vocational training).

*Combinations of employments possible.

Data are presented as median (IQR) for age and BMI and as n (%) for other variables.

Significant differences in bold font (Mann-Whitney U-Test for age and BMI, Chi-square test for employed, unemployed and student subcategories, Fisher’s exact test for other variables; *p < 0.05)*. Bonferroni test was applied when comparing subcategories of demographic variables between groups *(p < 0.01*).

Among the psychosocial assessments, GCPS, JFLS-20, GAD-7, and WPI scores exhibited significant differences between participants from the two cohorts. The GCPS-level IV, considered severely limiting, was present in 47.7% of Swedish participants compared to 13.5% of the Brazilian participants, while for level II frequencies were 17% vs. 39%, respectively. In the Swedish cohort, five participants met the criteria for myogenous TMD and reported pain during clinical examination. However, they did not indicate current pain or pain during the previous month in the questionnaire used to calculate GCPS and were therefore classified as GCPS Grade 0. There were no significant differences between both countries regarding CPI, PHQ-9, PHQ-15 and OBC scores (Table 2).

**Table 2:** Psychosocial assessments according to DC/TMD Axis II of the overall sample.

		Brazil *n* = 141	Sweden *n* = 159	*p*-value	Effect size
Pain interference (GCPS)				< 0.001	0.409
0		0 (0)	5 (3.2)	0.062	0.123
I		42 (29.8)	30 (18.8)	0.030	0.128
II		55 (39)	27 (17)	**< 0.001**	**0.247**
III		25 (17.7)	21 (13.2)	0.335	0.062
IV		19 (13.5)	76 (47.8)	**< 0.001**	**0.368**
Characteristic pain intensity		53.3 (23.4)	60 (40.0)	0.060	0.127
Jaw functional limitation scale		1.0 (2.1)	1.4 (2.6)	**0.027**	**0.147**
Depression (PHQ-9)		7.0 (7.0)	9.0 (8.0)	0.452	0.050
Anxiety (GAD-7)		9.0 (8.0)	6.0 (8.5)	**< 0.001**	**-0.265**
Physical symptoms (PHQ-15)		9.0 (6.0)	9.0 (7.5)	0.944	0.004
Oral behavior checklist		29.0 (12.0)	29.0 (14.0)	0.888	0.009
Widespread pain		9.0 (6.4)	23.0 (14.5)	**0.024**	**0.131**

GCPS: Graded Chronic Pain Scale.

Data are presented as n (%) for GCPS and widespread pain and as median (IQR) for other variables. Significant differences in bold font (Chi-square test for Widespread pain, Fisher’s exact test for GCPS, Mann-Whitney U-Test for other variables; *p < 0.05)*. Bonferroni test was applied when comparing subcategories of GCPS between groups *(p < 0.01)*.

Table 3 shows the number and frequency of TMD subtypes and headache attributed to TMD for both cohorts. There were significant differences in the distribution of the TMD diagnoses. Differences were not significant for myalgia, disc displacement without reduction without limited opening, and headache attributed to TMD. Most of the participants in both countries had more than one type of TMD, which was observed in 80.8% of Brazilians and in 91.2% of Swedes.

**Table 3:** TMD diagnoses according to the DC/TMD criteria. Several diagnoses possible. Data show as n (%).

		Brazil *n* = 141	Sweden *n* = 159	*p*-value	Effect size
Pain-related TMDs					
Myalgia		67 (47.5)	85 (53.5)	0.304	0.059
Myofascial pain with referral		74 (52.5)	74 (46.5)	0.304	0.059
Mixed TMD (myalgia + arthralgia)		71 (50.4)	113 (71.1)	**< 0.001**	**0.212**
Headache attributed to TMD		77 (54.6)	87 (54.7)	0.985	0.001
Intra-articular TMDs					
DD with reduction		9 (6.4)	36 (22.6)	**< 0.001**	**0.227**
DD with reduction with intermittent locking		0 (0)	21 (13.2)	**< 0.001**	**0.258**
DD without reduction with limited opening		20 (14.2)	6 (3.8)	**0.002**	**0.185**
DD without reduction without limited opening		15 (10.6)	9 (5.7)	0.113	0.091
Degenerative joint disease		2 (1.4)	33 (20.8)	**< 0.001**	**0.301**

DC/TMD: Diagnostic Criteria for Temporomandibular Disorders; DD: Disk displacement.

Significant differences in bold font (Fisher’s exact test for DD with reduction with intermittent locking and Degenerative joint disease, Chi-square test for other variables; *p < 0.05)*.

## Discussion

Psychosocial symptoms in TMD patients are well-recognized in the literature, with several studies showing an association between TMD pain and disorders such as depression, somatization, and anxiety^31^. Cross-country comparisons are essential to understand how socioeconomic context and cultural factors affect the biopsychosocial mechanisms underlying TMD and its management. However, to the authors’ knowledge, few studies have compared psychosocial and demographic factors of women with TMD from two countries with different socioeconomic development levels^14,32^. In this investigation, significant differences between the cohorts from Sweden and Brazil were observed regarding GCPS, JFLS-20, GAD-7, WPI and sociodemographic aspects. Therefore, the null hypothesis was rejected.

It is well established that TMDs are complex conditions with multifactorial etiology, aligning with the biopsychosocial model of illness^3^. In this context, psychosocial distress, environmental contributions, and demographic variances may play a significant role in the development, presentation, and treatment of chronic pain conditions, such as TMD^3,33^. Our findings pointed to significant demographic differences in both countries regarding BMI, marital status and education level.

Although a recent systematic review suggested that higher BMI may act as a protective factor of TMD^34^, other studies reported conflicting results, revealing that pain-related TMD symptoms were significantly more frequent among those who were overweight^35,36^. In the present sample, Brazilian participants showed a higher BMI (25 kg/m^2^), i.e. classified as overweight, compared to the Swedish population (23 kg/m^2^), which is within the normal range^37^. These findings were similar to the global status report of the World Health Organization^38^. Nevertheless, the difference was small and different methods for assessing the BMI in the Swedish and Brazilian sample may have influenced the results. In Sweden, participants self-reported their height and weight, whereas in Brazil, these measurements were obtained through direct evaluation. It is well known that self-report is less reliable than actual measurement, especially for weight, which people tend to report lower than actual^39^.

Marital status was also a variable with significant differences between the two samples, notably with higher percentage of single women in Brazil (65.2%) compared to Sweden (21.4%). TMD has been associated with married status and TMD-related chronic pain^40^. A previous study reported that the estimated risk of developing TMD was 2.8 times higher for single individuals^11^. However, the role of this factor in TMD remains limited^41^. Previous studies also revealed that lower educational levels may be related to more intense TMD symptoms^42^. Our findings support this hypothesis, since the Swedish cohort presented higher levels of GCPS and lower educational levels compared to Brazilian cohort.

The prevalence of TMD is three times higher in women, with peak of incidence between 20 and 40 years of age^43,44^. Our sample was composed of women only; therefore, gender-related differences were not assessed. Regarding age, no significant differences were observed between the Brazilian and Swedish groups, with mean ages of 30.5 and 30.6 years, respectively. These findings are consistent with previous sociodemographic studies, which report a peak of TMD prevalence in the ages 30 to 40 years^45,46^.

All participants included in this retrospective study were diagnosed with myalgia, the most prevalent TMD condition^47^, using the instruments from Axis I of the DC/TMD^4^. In Brazil, 54.6% of the cohort was diagnosed with headache attributed to TMD, followed by myofascial pain with referral (52.5%) and mixed TMD (50.4%). In contrast, in Sweden, 71.1% presented with mixed TMD, followed by headache attributed to TMD (54.7%), and myalgia without associated arthralgia (53.5%). The higher frequency of myalgia compared with articular disorders found in both countries is in accordance with the literature^48^. Except for myalgia, myofascial pain with referral, disc displacement without reduction without limited opening, and headache attributed to TMD, most subdiagnoses differed significantly between the two countries. The observed disparities between the groups may be explained by variations in healthcare systems, referral pathways, recruitment procedures, and patient characteristics across the two countries. Although the same diagnostic criteria (DC/TMD) were used, differences in clinical settings, study populations, and the absence of inter-examiner calibration may also have affected the diagnostic distribution. Moreover, these findings may reflect the multifactorial etiology of TMD, in which socioeconomic conditions, environmental exposures, and psychosocial factors differentially influence its signs and symptoms^3,49^.

Axis II of DC/TMD provides valuable instruments for the assessment of pain intensity, pain-related disability, jaw function, psychosocial distress, parafunctional behaviors, and widespread pain^22^. We found a significant difference in GCPS, JFLS-20, GAD-7 and widespread pain between the countries. In Sweden, GCPS and JFLS-20 were higher compared to individuals from Brazil. These results may be attributed to differences in recruitment sources, as patients referred to specialist clinics tend to exhibit more severe symptoms of TMD^50^. Also, Swedish participants more often reported presence of widespread pain compared to the Brazilian participants. This finding may also help to explain the higher pain interference in the Swedish cohort, as previous research has demonstrated a positive correlation between widespread pain, higher grades of pain interference, comorbid pain conditions, and TMD pain^51^. In addition, cold exposure may contribute to the higher prevalence of widespread pain in the Swedish cohort, as some studies suggest that it may increase the risk of musculoskeletal disorders^52^ and contribute to the occurrence of pain across multiple body sites^53,54^.

Among all the psychosocial variables investigated in this study, only GAD-7 differed between groups, with higher values in Brazilian individuals. This condition is often associated with TMD^43,46^ and, according to a recent epidemiological study, Brazil ranks as the second country with the highest levels of anxiety on a world scale^55^. This study also revealed that the prevalence of anxiety disorders appears to increase with socioeconomic development, a higher dependent older population, and urbanization^55^. Although Brazil is not considered a developed country, the high prevalence of anxiety may be explained by a combination of factors, including rapid urbanization, lifestyles resembling those of wealthier countries, internal social and economic pressures, and improved detection and reporting of cases compared with other developing regions. Moreover, alcohol consumption was also correlated with more symptoms of anxiety^55^.

Regarding OBC, there was no statistically significant difference between cohorts. However, the median OBC scores in both Brazil^29^ and Sweden^29^ are considered high and also a risk factor for TMD onset^22^.

Overall, our findings showed differences in psychosocial and demographic characteristics between the countries but also some similarities between the cohorts. These similarities suggest that, despite socioeconomic and cultural variations, the biopsychosocial mechanism of TMD may be universal across populations.

Since this was a retrospective study, several limitations should be addressed. These include e.g. different ways of assessing variables, such as WPI vs. pain drawings, self-report of weight and height vs. on-site measurements that may have influenced the results. However, both sites used the DC/TMD axis I and II, which is a strength and enable direct comparison between cohorts. Nevertheless, two centers with several examiners participated in this study. Although they were calibrated according to reference standard examiners, this may have introduced a risk of diagnostic bias, as seen in the articular TMD diagnoses, and lack of homogeneity across centers. Finally, the inclusion of only women makes the generalizability of the findings questionable, even if the majority of TMD patients are women^3,4,43^. Further studies, including participants of both sexes and a broader range of countries are needed to better understand the differences in psychosocial distress and demographic characteristics of TMD in different populations.

## Conclusion

There were some differences and some similarities between TMD patients in the two centers in Brazil and Sweden. The comprehensive assessment of TMD diagnoses, psychosocial and demographic characteristics may help to guide TMD care and future research endeavors in countries with different socioeconomic development. However, future research is needed to better understand the context of TMD in different populations.

## Data Availability

The datasets used and/or analysed during the current study are available from the corresponding author on reasonable request.

## Abbreviations

TMD: Temporomandibular disorders
BMI: Body mass index
DC/TMD: Diagnostic criteria for temporomandibular disorders
GCPS: Graded chronic pain scale
CPI: Characteristic pain intensity
DP: Disability points
WPI: Widespread pain index
GAD-7: Generalized anxiety disorder-7
PHQ-9: Patient health questionnaire-9
PHQ-15: Patient health questionnaire-15
JFLS-20: Jaw functional limitation scale
OBC: Oral behaviors checklist
